# True brachial artery aneurysm secondary to arterial wall infiltration by Langerhans cell histiocytosis

**DOI:** 10.1016/j.jvscit.2026.102500

**Published:** 2026-09-04

**Authors:** Pietro Federico Ricciardi, Aurore Roesti, Chiara Saglietti, Laurence de Leval, Marie Nicod Lalonde, Celine Deslarzes-Dubuis

**Affiliations:** a Vascular Surgery Unit, Heart and Vessels Department, Lausanne University Hospital, Lausanne, Switzerland; b Institute of Pathology, Department of Laboratory Medicine and Pathology, Lausanne University Hospital and Lausanne University, Lausanne, Switzerland; c Nuclear Medicine and Molecular Imaging, Department of Medical Radiology, Lausanne University Hospital, Lausanne, Switzerland

**Keywords:** Multifocal Langerhans cells histiocytosis, Humeral artery aneurysm, Arterial infiltration, Vascular reconstruction, Positron emission tomography/computed tomography

## Abstract

Langerhans cell histiocytosis is a rare histologic disorder that can affect and damage any tissues and organ in the body. A 40-year-old man with multisystem Langerhans cell histiocytosis presented with a painful distal arm mass. Imaging demonstrated a 3.6-cm true distal brachial artery aneurysm and progressive hypermetabolic activity of the affected artery on the retrospective review of serial positron emission tomography/computed tomography scans. The patient underwent aneurysm resection and arterial reconstruction using a reversed great saphenous vein bypass. Histopathology confirmed infiltration of the intima and media by Langerhans cells with destruction of the elastic lamina. Subsequent disease-specific therapy achieved a complete metabolic response, and vascular reconstruction remains patent.

First described by Paul Langerhans in 1868, Langerhans cells are dendritic cells normally located in the epidermis as well as in the oral and genital mucosa, where they play a key immunological role. They characteristically express CD1a and CD207 (langerin) and contain Birbeck granules on immunohistochemical and ultrastructural examination.^1^^,^^2^

Pathological proliferation and tissue infiltration of these cells result in Langerhans cell histiocytosis (LCH), a rare entity belonging to the group of histiocytic neoplasms. The pathophysiology of LCH is driven by activating mutations in the mitogen-activated protein kinase (MEK) signaling pathway, most commonly involving *BRAF V600E* or *MAP2K1*, but also other upstream components such as *NRAS*, *KRAS*, *HRAS*, and *PTPN11*.^3^^,^^4^

Clinical presentation is heterogeneous and largely depends on the sites involved. Manifestations may result from tumoral mass effects and compression of adjacent structures, inflammation, tissue destruction, progressive fibrosis, or a combination thereof. LCH is classified as single-system or multisystem disease, with bone and skin being the most frequently affected organs. Involvement of the liver, spleen, or bone marrow is associated with a more aggressive disease course.^1^, ^2^, ^3^, ^4^

Arterial involvement is exceptionally rare. To date, only isolated cases have been reported including external carotid artery pseudoaneurysm caused by head and neck LCH invasion,^5^ a stroke caused by intracranial artery stenosis in a young patient with multifocal LCH^6^ and a case of cardiovascular involvement presenting as right atrial and ventricular wall thickening as well as the involvement of aortic arch and descending aorta.^7^ Given the potentially catastrophic consequences, early recognition of vascular involvement is crucial to allow timely referral for specialized care.

Diagnosis relies on histopathological confirmation supported by radiological findings. In recent years, targeted therapies—including BRAF, MEK, tropomyosin receptor kinase, and mechanistic target of rapamycin inhibitors—have profoundly changed the management and salvage treatment of patients with refractory or multisystem disease.^3^

Written informed consent was obtained from the patient for publication of this case report.

## Case report

A 40-year-old man with end-stage renal disease secondary to congenital anomalies of the kidney and urinary tract required hemodialysis through a left radiocephalic arteriovenous fistula (AVF) from 2010 to 2016 and subsequently underwent kidney transplantation in August 2016. In April 2018, his AVF was surgically closed.

In February 2022, he was diagnosed with multifocal osseous LCH following biopsy of an epidural mass at the C7 to T1 level, which required radiotherapy and bisphosphonate therapy. Follow-up positron emission tomography (PET)/computed tomography (CT) demonstrated disease progression with the appearance of diffuse visceral and iliofemoral lymphadenopathy (Fig 1, *A*). Biopsy of an inguinal lymph node confirmed LCH (*BRAF-V600* negative) with a *MAP2K1* gene mutation in exon 3. Retrospective review of serial PET-CT scans demonstrated preoperative progressive focal hypermetabolism of the distal left brachial artery (Fig 1, *A-F*).

**Fig 1:**
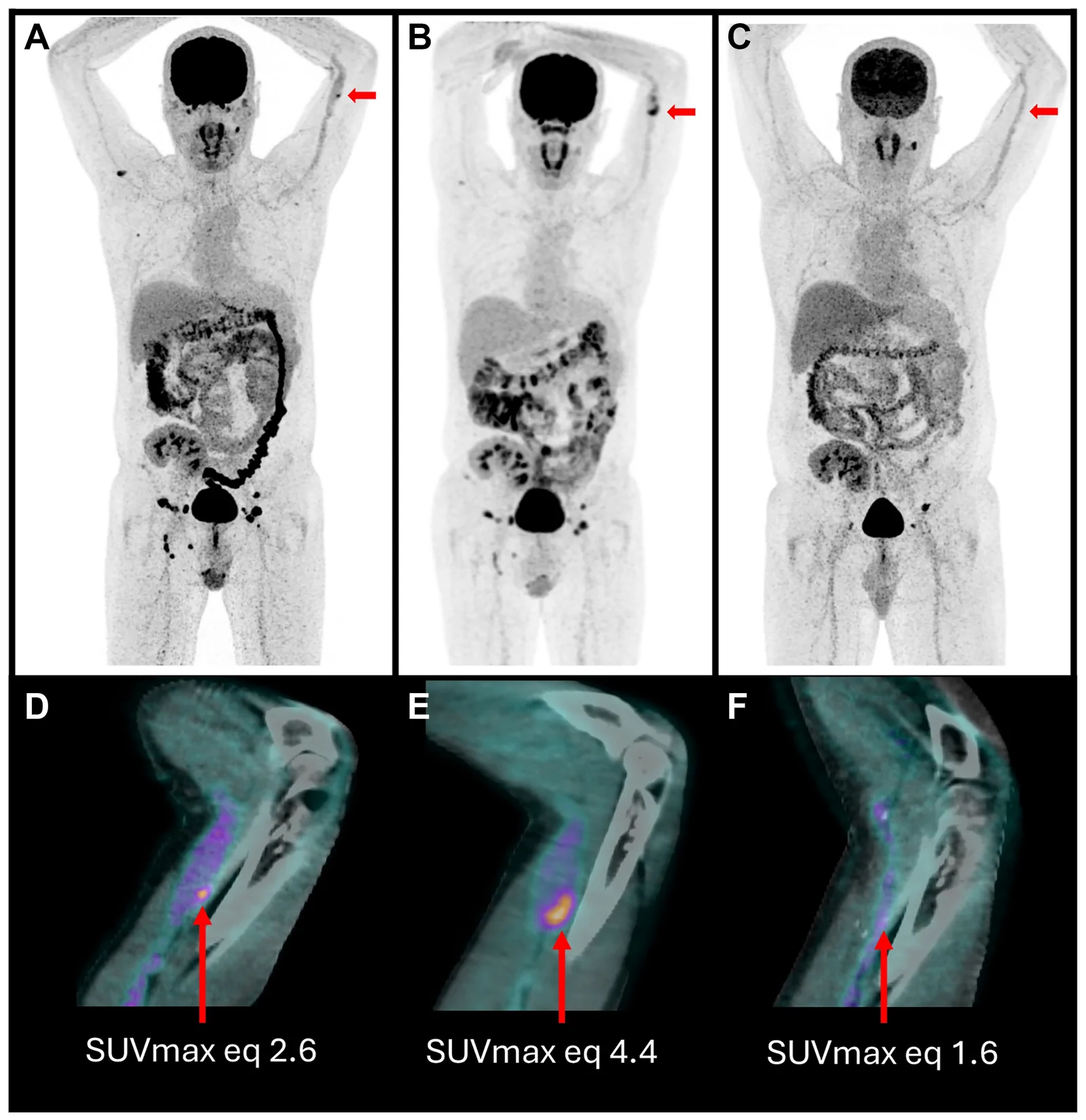
Whole-body positron emission tomography (PET)/computed tomography (CT) maximum intensity projection (MIP) images showing focal fluorodeoxyglucose (FDG) uptake along the course of the left brachial artery (*red arrow*) in September 2022 **(A)**, with increased uptake in May 2023 **(B)**, and complete resolution at the most recent follow-up in May 2026 **(C)**. Corresponding fused PET/CT images localize the FDG uptake to the left brachial artery (*red arrow*) in September 2022 **(D)** and May 2023 **(E)**, with no residual uptake visible at follow-up in May 2026 **(F)**. SUVmax eq: equivalent maximum standardized uptake value, adjusted to account for differences between PET/CT systems.

In May 2023, the patient presented to the emergency department with a painful mass of the distal left arm, just proximal to the elbow. Duplex ultrasound revealed an aneurysm of the left brachial artery associated with asymptomatic distal embolization to the radial artery (Fig 2, *A* and *B*). CT angiography confirmed a distal brachial artery aneurysm measuring 33 × 36 and 55 mm in length (Fig 2, *C* and *D*).

**Fig 2:**
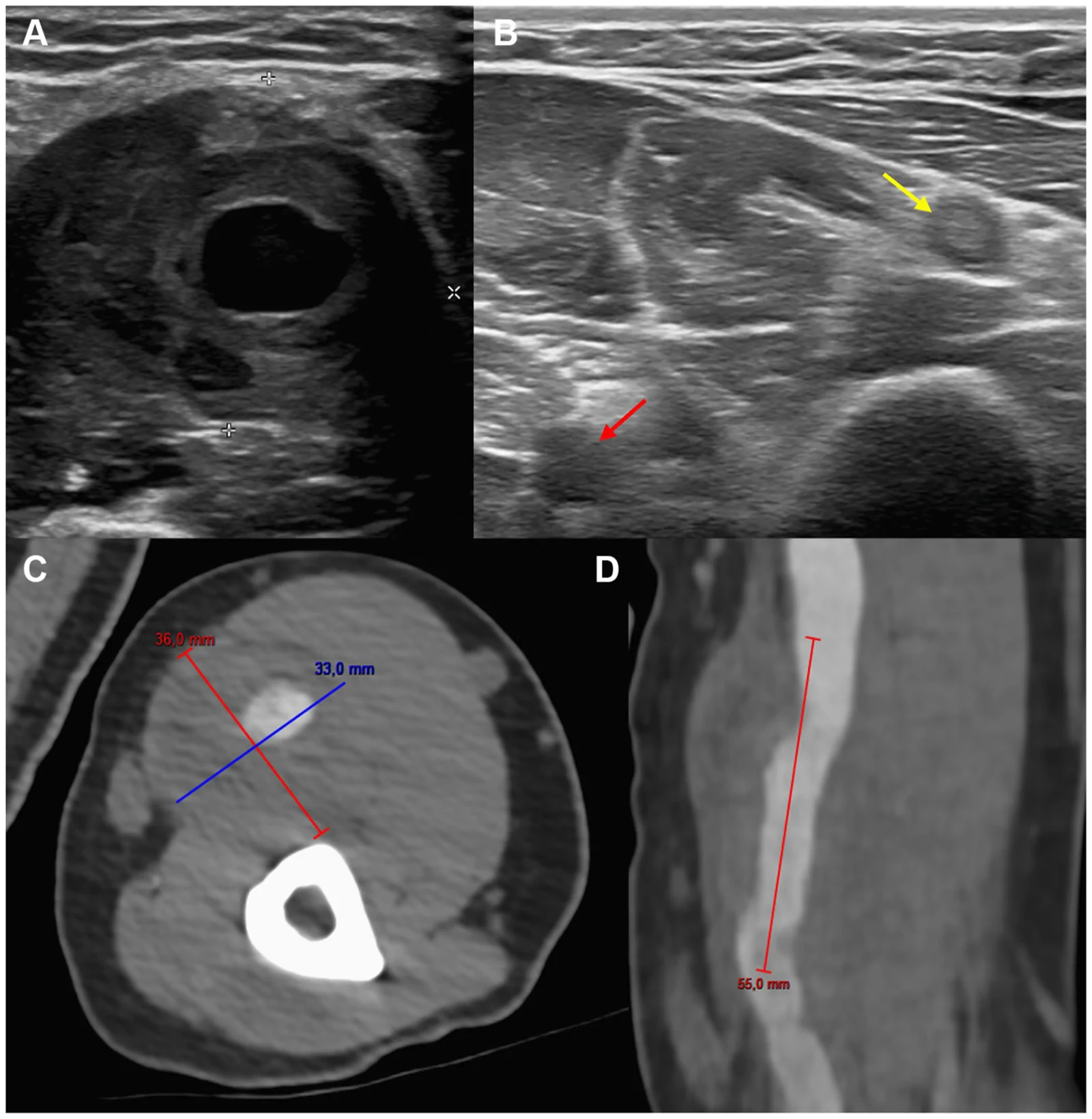
Radiological findings: duplex ultrasound image of the brachial artery aneurysm **(A)**, radial artery with thrombus (**B,***yellow arrow*) and ulnar artery patent (**B,***red arrow*); computed tomography angiography images of the brachial artery aneurysm, measuring 33 × 36 mm on axial plane **(C)** and 55 mm in length on sagittal plane **(D)**.

Given the symptomatic aneurysm and distal embolization, we introduced an anticoagulation therapy with low-molecular-weight heparin, and a surgical treatment was performed on June 13. A polylobulated aneurysm of the distal brachial artery was resected, followed by arterial reconstruction using an interposition graft with reversed left great saphenous vein. In the presence of adequate hand perfusion, with a palpable ulnar pulse and a first finger plethysmographic pressure of 110 mmHg, the patient was treated with prophylactic antithrombotic therapy during hospitalization, and aspirin 100 mg once a day was initiated for 1 year. Owing to clear aneurysmatic origin to the radial embolization, no further investigation was conducted.

Gross examination of the resected brachial artery specimen revealed a markedly thickened and remodeled arterial wall, with the lumen partially occluded by a mural thrombus. After pathological fixation, the ex vivo maximal diameter was of 2.8 cm (Fig 3).

**Fig 3:**
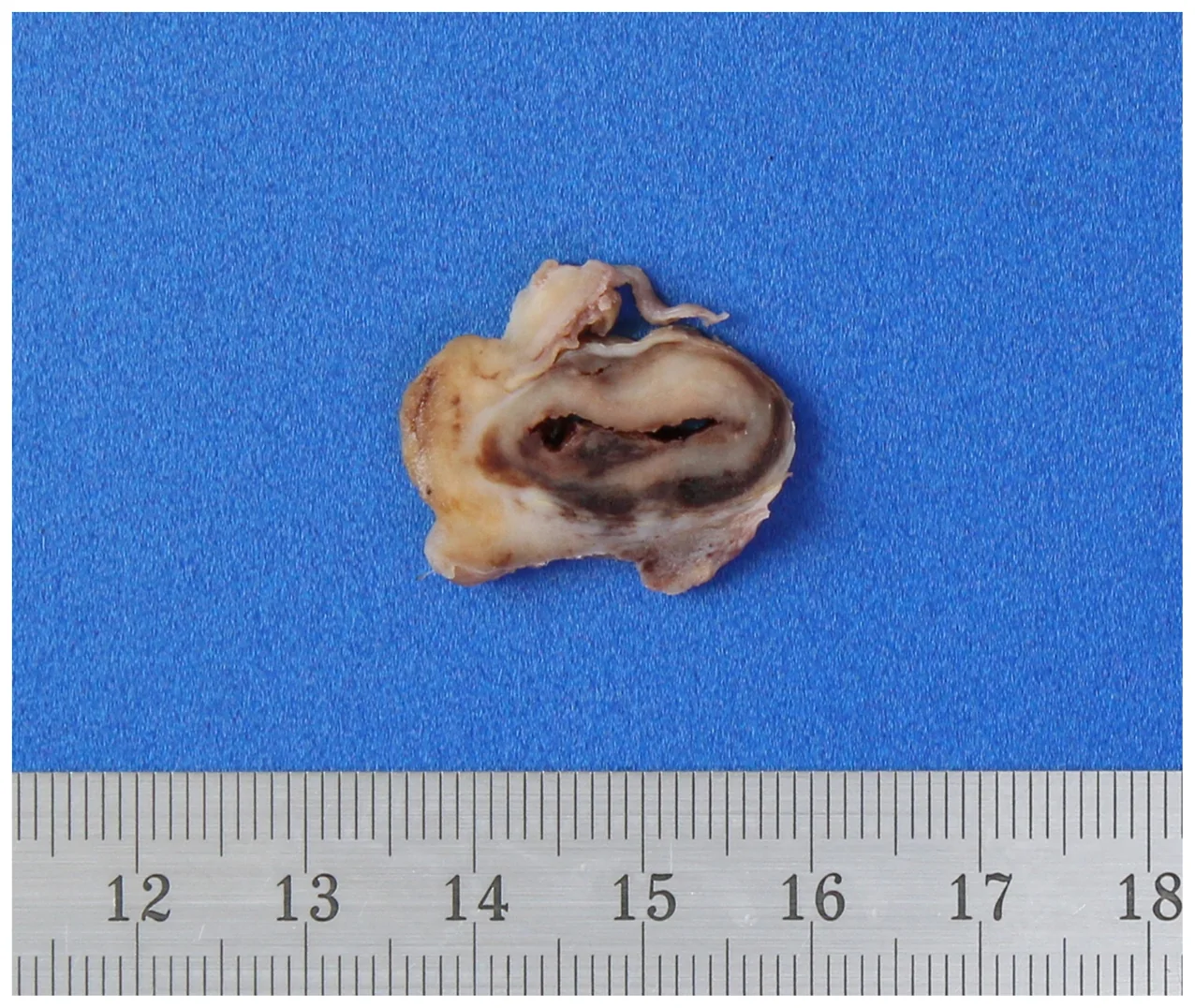
Cross-sectional view of the brachial artery aneurysm, measuring ex vivo up to 2.8 cm in diameter.

Hematoxylin and eosin staining demonstrated diffuse infiltration of the intimal and medial layers by Langerhans cells associated with numerous eosinophils, as well as an aneurysmal dilatation with an occlusive mural thrombus (Fig 4, *A* and *B*). Verhoeff-Van Gieson elastic staining confirmed infiltration of the arterial wall with partial destruction of both the internal and external elastic laminae (Fig 4, *C*). Immunohistochemical analysis showed diffuse expression of CD1a and S100 by the infiltrating Langerhans cells (Fig 4, *D* and *E*), confirming direct arterial involvement by LCH.

**Fig 4:**
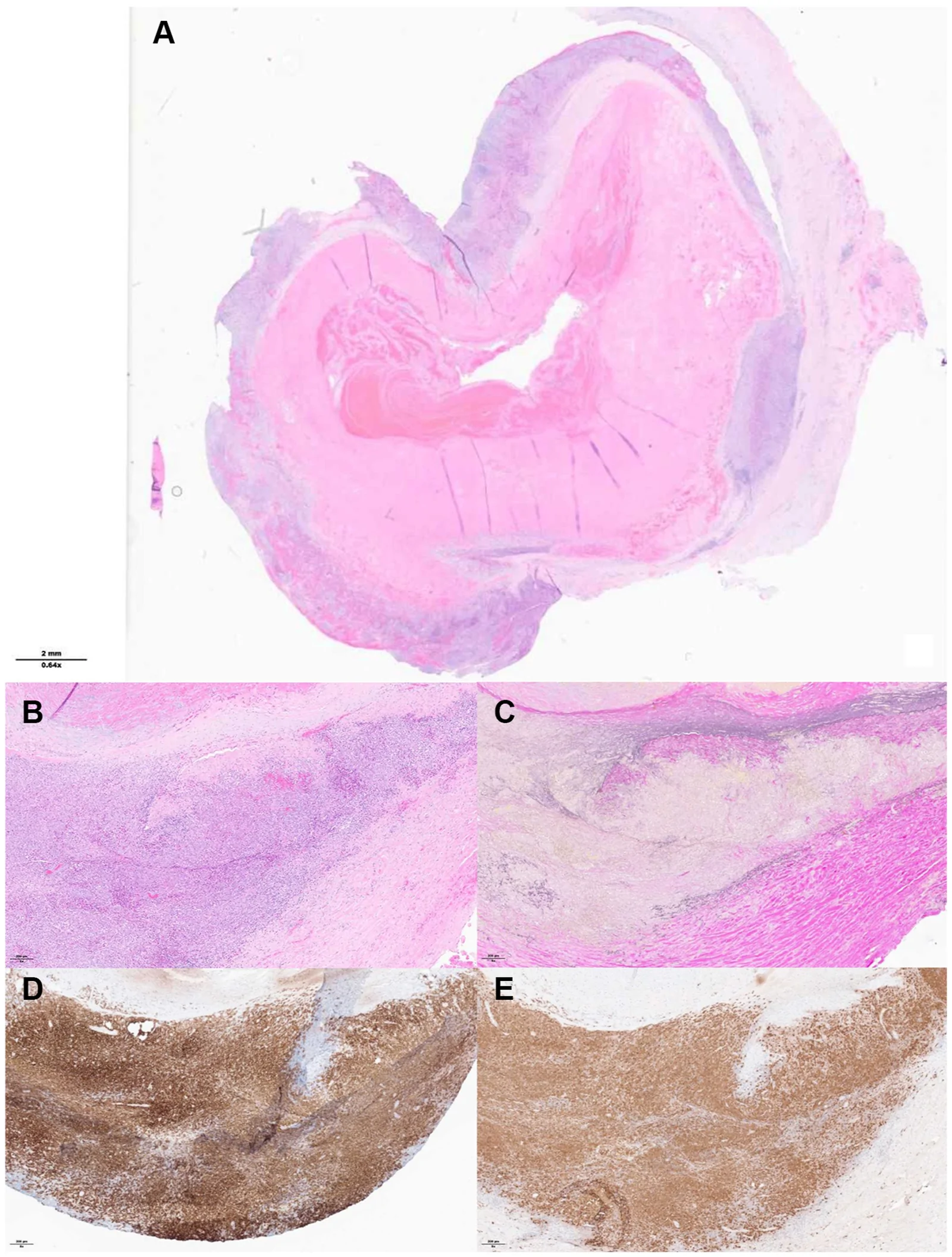
Hematoxylin and eosin (H&E) staining at 2 mm, ×0.64 magnification **(A)** showing diffuse infiltration of the intima and media by neoplastic Langerhans cells, associated with an occlusive mural thrombus. Higher magnification at 200 μm, ×5 **(B)** reveals characteristic Langerhans cell proliferation admixed with numerous eosinophils. Verhoeff-Van Gieson (VVG) staining at 200 μm, ×5 magnification **(C)** shows disruption of the internal and external elastic laminae. Immunohistochemical stain panels at 200 μm, ×5 magnification of CD1a protein **(D)** and S100 protein **(E)** supporting Langerhans cell differentiation.

Following multidisciplinary discussion, cytarabine chemotherapy was initiated for multisystem disease. Subsequent PET-CT demonstrated disease progression with osseous involvement, requiring a radical right mastoidectomy in August 2024, which confirmed histiocytic infiltration. Oncologic treatment was therefore escalated to a newer, more selective molecule, cobimetinib fumarate, a MEK inhibitor, resulting in a complete metabolic response and regression of hypermetabolic lesions on follow-up PET-CT.

At the 36-months follow-up, on last duplex ultrasound in June 2026, we confirmed graft patency without stenosis or recurrent embolic event, and no ischemic hand symptoms.

## Discussion

LCH is a rare clonal histiocytic disorder with highly heterogeneous clinical manifestations. Although bone, skin, lymph nodes, and visceral organs are commonly affected, arterial involvement remains exceptionally rare. To date, only isolated vascular cases have been reported, as summarized in Table.

**Table:** Summary of published case reports describing vascular involvement in Langerhans cell histiocytosis (*LCH*)

Clinical manifestation	Proposed pathophysiological mechanism	Evidence	Authors
External carotid artery pseudoaneurysm	Infiltration and invasion from head and neck LCH	Histological	Herman et al^5^
Acute ischemic stroke caused by intracranial artery stenosis	Immunological with possible intracranial periarterial inflammatory infiltration	Radiological	Zhang et al^6^
Right atrial and ventricle, aortic arch, and the descending aorta wall thickening	Imaging findings consistent with vascular LCH infiltration	Radiological	Talarico et al^7^
True humeral artery aneurysm	Direct infiltration of intima and media layer by LCH causing structural weakening	Histological	Present case

However, the patient’s history of a previous ipsilateral radiocephalic AVF should be considered as a potential contributing factor of brachial aneurysm development due to chronic hemodynamic stress, even after ligation.^8^

In this case, histologically confirmed direct infiltration of the arterial wall by LCH strongly supports a mechanism of progressive wall weakening leading to true aneurysm formation. Recognition of this rare manifestation is important because delayed diagnosis may expose patients to thromboembolic, ischemic, or hemorrhagic complications.

An additional noteworthy finding was the progressive focal hypermetabolism of the affected brachial artery, identified retrospectively on serial PET-CT examinations before the onset of vascular symptoms. Although the significance of vascular hypermetabolism in LCH remains uncertain, this observation suggests that PET-CT may help detect early arterial involvement in patients with aggressive or multisystem disease.

The patient’s history of kidney transplantation and chronic immunosuppression may have contributed to the atypical disease course, although a causal relationship cannot be established. Whether immunosuppression influences the risk of vascular involvement in LCH remains unknown, as no specific data addressing this association are currently available. Nevertheless, extrapolating from previous reports in immunocompromised patients, including liver transplant recipients, altered immune surveillance has been hypothesized to favor uncontrolled clonal proliferation of histiocytic disorders, potentially contributing to more aggressive or disseminated disease manifestations.^9^^,^^10^ This hypothesis remains speculative and warrants further investigation.

## Conclusions

This case broadens the spectrum of vascular manifestations associated with LCH and demonstrates that direct arterial infiltration may lead to true aneurysm formation. Early recognition and multidisciplinary management combining vascular surgery and disease-specific systemic therapy are essential to prevent potentially limb-threatening complications.

The favorable response to MEK inhibition in our patient raises the question of whether earlier targeted therapy could be considered in selected patients with multisystem LCH harboring mitogen-activated protein kinase pathway mutations.

## Funding

The authors received no financial support for the study design; data collection, analysis, and interpretation; manuscript writing; the decision to submit the article; and the creation of the manuscript.

## Disclosures

The authors have no competing interests.
